# On Crashworthiness and Energy-Absorbing Mechanisms of Thick CFRP Structures for Railway Vehicles

**DOI:** 10.3390/polym14224795

**Published:** 2022-11-08

**Authors:** Dongdong Chen, Xiaoyu Sun, Benhuai Li, Yanwen Liu, Tao Zhu, Shoune Xiao

**Affiliations:** 1 State Key Laboratory of Traction Power, Southwest Jiaotong University, Chengdu 610031, China; 2 Basic R&D Department, National Railway Passenger Car Engineering Research Center, Changchun Railway Vehicles Co., Ltd, Changchun 130113, China

**Keywords:** CFRP, impact test, energy-absorbing mechanisms, numerical simulation, crashworthiness design

## Abstract

This study aims to provide important guidelines for the crashworthiness design of composite energy-absorbing structures, especially railway vehicles. An experimental and numerical investigation was carried out to explore the crushing response of circular composite tubes reinforced with plain woven carbon fiber-reinforced polymers (CFRP). Quasi-static and dynamic axial crushing tests were performed on CFRP tubes with an inner diameter of 100 mm and a nominal wall thickness of 12 mm. Experimental results showed that increasing loading velocity led to a 21.8% reduction in specific energy absorption (from 99.7 kJ/kg to 78.7 kJ/kg) but had negligible influence on failure modes. Finite element models were also established and validated against the experimental results using ABAQUS/Explicit software. The effects of several different parameters such as the number of shell layers, friction coefficient, and interface properties on the simulated results, were also investigated and analyzed. A small variation in these parameters could change the total energy absorption of CFRP tubes. The comparisons between the predicted and experimental results indicated that a finite element model with 10 shell layers could effectively replicate the crushing response. In addition, the simulated results indicated that the damage of tubal wall materials dominated the major energy-absorbing mechanisms of CFRP tubes under quasi-static loads, which was 69.1% of the total energy. The energy dissipated by friction effects between the loading platen and the crushed fronds was 24.1% of the total energy. The increase in the loading velocity led to a decrease in the composite damage energy except for friction energy, resulting in a decrease in the total energy absorption.

## 1. Introduction

Thin-walled structures have excellent advantages, such as high mechanical performance, ease of forming, and lightweight efficiency [1,2,3]. One of their typical applications is in energy-absorbing components, such as crash boxes, frontal beams for automobiles, and anti-climbers for railway vehicles [1,3,4]. During vehicle collision accidents, kinematic energy is dissipated through a controlled deformation process of these structures, and the safety of passengers can thus be guaranteed. The ability of these structures to convert kinematic energy into internal energy and protect the occupants from accidents is often referred to as crashworthiness [5]. Compared to their metallic counterparts, composite structures, especially those made from plain woven carbon fiber-reinforced polymer (CFRP), often show excellent advantages such as higher energy absorption efficiency, better mechanical properties, and excellent resistance to corrosion, fatigue, and creep [1,6]. However, owing to their anisotropic material properties, the crashworthiness design of CFRP structures is a complicated process that has attracted considerable attention in recent decades.

Numerous experimental studies have been conducted to explore the energy-absorbing mechanisms of thin-walled CFRP structures under axial crushing loads [7,8,9,10]. Researchers have found that three distinctive failure modes exist in these structures: progressive end-crushing, unstable local buckling, and mid-length collapse [7,8]. The progressive end-crushing mode, which comprises a combination of progressive splaying and transverse shear, leads to a higher load-carrying capacity than other modes. In [9,10], the authors identified that the total impact energy can be dissipated with the transverse shearing in CFRP fronds (approximately 65%), friction between the loading platen and CFRP fronds (approximately 30%), and crack propagation. Generally, these failure mechanisms are sensitive to various design factors, such as fiber/epoxy types [11,12,13,14], stacking configuration [15,16,17,18], dimensions [19,20], cross-section shapes [21,22,23], and triggers [23,24]. Several reviews have also appeared on the crashworthiness of composite energy-absorbing tubes [1,3,4,25]. Moreover, considering the characteristics of accidents involving automobile collisions, the crushing behavior of CFRP structures subjected to different impact velocities was investigated. Owing to the differences in the sensitivity of the above-mentioned design factors to strain rates, the reported conclusions on the effect of an increase in loading velocity on the energy absorption of CFRP structures mainly fall into the following categories: 14.8–26% increase [19,26,27], up to 61% decrease [17,18,28,29], and negligible influence [27,30]. There is strong interest in investigating the energy-absorbing mechanisms and crashworthiness of CFRP structures under dynamic axial crushing loads.

Compared to experimental tests, finite element (FE) simulation presents excellent advantages in reducing the test cost and understanding the energy-absorbing mechanisms. Compared to solid elements, shell elements have a comparable accuracy but higher efficiency and have been widely adopted in the crashworthiness simulation of composite structures using commercial FE software such as ABAQUS [31,32,33,34], LS-DYNA [35,36,37,38,39], ESI-VPS [40], and PAM-CRASH [41,42]. The stacked-shell modeling strategy, in which the intra-layer and inter-layer deformation behavior is modeled via several shell layers and cohesive contacts/elements inserted, has been widely adopted to simulate the progressive crushing behavior of composite structures. Researchers have found that the predicted results are sensitive to various factors, such as the stress–strain relationship (constitutive models) [43,44,45], number of shell layers, and friction coefficient [31,46]. However, the computational cost of the stacked-shell models increased significantly after the structural dimensions were enlarged. Recently, Song et al. [47] introduced a novel equivalent method for simulating the crashworthiness of CFRP structures. Results show that the computational efficiency can be improved by 62–86% compared to the traditional stacked-shell models.

However, most existing studies focus on the crashworthiness of CFRP structures with a wall thickness of less than 5 mm and a maximum mean crushing force of less than 100 kN [1,3]. In practice, energy-absorbing structures with a larger thickness and load-carrying capability are required in the railway industry. For example, railway vehicles have a larger mass than automobile equipment, which often leads to higher collision energies [48,49]. These energy-absorbing components are often required to carry a maximum average impact force of 500–2000 kN with wall thicknesses of 7–12 mm [50,51,52]. Conclusions from existing studies have shown that the failure mechanisms and mechanical performance of thick composite panels (with thicknesses larger than 6 mm) differ from those of thinner panels [53,54,55]. There is a great significance to investigating the energy-absorbing mechanisms and crashworthiness performance of CFRP structures with large load-carrying capabilities.

In this study, experimental and numerical approaches were adopted to investigate the energy-absorbing mechanisms of circular CFRP tubes with a [0/90]_60_ layup. Quasi-static and dynamic axial impact tests were performed to obtain the typical crushing response, including force–displacement curves, crushing failure mode, and crashworthiness parameters. Axial crushing FE models were established and validated against the experimental results. In addition, the effects of the number of shell layers, friction coefficient, and interface properties were investigated and analyzed in detail. Finally, the effects of the loading velocity on the energy-absorbing mechanisms of the CFRP structures were compared and analyzed.

## 2. Experimental Testing and Results

### 2.1. Specimen Preparation

**Figure 1:**
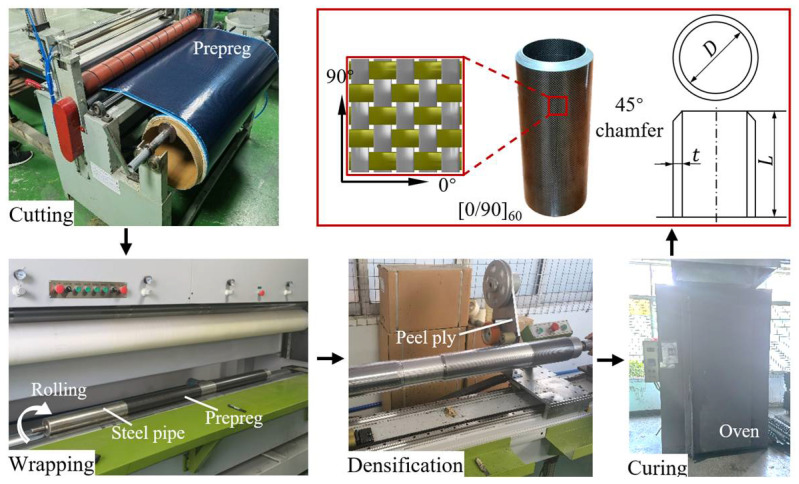
Fabrication process of circular CFRP tubes.

A circular-shaped tube is a commonly used component in the crashworthiness design of railway vehicles, such as the energy-absorbing device [50,51,56]. As shown in Figure 1, the circular CFRP tubes were designed with an inner diameter (*D*) of 100 mm and a length (*L*) of 310 mm. Specimens with a [0/90]_60_ layup were then fabricated using a plain woven T300/3K carbon fiber/epoxy prepreg supplied by Guangdong Hangyu Composite Material Technology Co. Ltd., Guangzhou, China. The area density of the fabrics used was 200 g/m^2^. The average diameter of the carbon fibers was 7 μm.

Figure 1 also presents the fabrication process of circular CFRP tubes, which comprises the following steps: (i) cutting the prepreg into pieces with proper dimensions, (ii) wrapping the prepreg around a circular steel pipe with a diameter of 100 mm, (iii) eliminating potential voids (densification) by overwrapping the stacked prepregs with a peel ply, (iv) curing in an oven at a temperature of 150 °C for 3 hours, and (v) cooling down and demolding. Finally, specimens with a thickness (*t*) of 12.4 mm are cut from the cured tubes.

### 2.2. Test setup

Table 1 summarizes the detailed specimen information and the axial crushing test conditions. CFRP-QS and CFRP-D indicate the CFRP specimens tested under quasi-static (QS) and dynamic (D) crushing loads, respectively. To eliminate the potentially unstable collapse mode caused by the high initial peak force, a 45° chamfer was machined on one side of each specimen (as illustrated in Figure 1).

**Figure 2:**
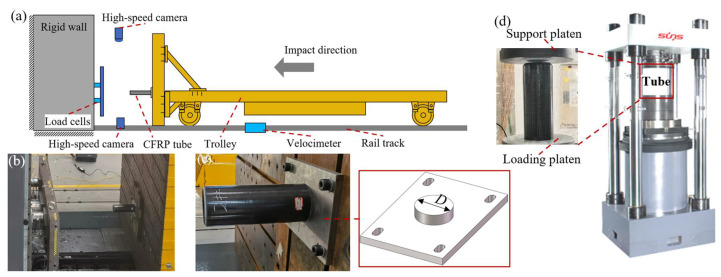
(**a**) Layout schematic, (**b**) collision zone, and (**c**) test trolley and specimen for dynamic impact test and (**d**) quasi-static test setup.

Dynamic crushing tests of the CFRP specimens were carried out at CRRC Changchun Railway Vehicles Co., Ltd., Changchun, China. Figure 2a presents the layout schematic, which consists mainly of a trolley, rigid wall, high-speed cameras, velocimeter, and rail track. During the test process, a trolley with a mass of 18.19 t was powered to an initial velocity of 1.944 m/s before coming in contact with the rigid wall along the railway track (Figure 2b). The specimen was installed on a trolley using a mounting device (Figure 2c). A velocimeter was used to obtain the actual impact speed of the trolley. High-speed cameras were placed at the top and side of the trolley to record the deformation and failure processes of the specimen. The instantaneous crushing force data were recorded by the load cells after the specimen contacted the platen, which was assembled on the rigid wall.

A quasi-static axial test was performed using a universal testing machine supplied by Shenzhen SUNS Technology Stock Co., Ltd., Shenzhen, China. As shown in Figure 2d, the specimen was placed freely between the loading and support platens before the test. A constant loading velocity of 0.00108 m/s was used during the testing process. The instantaneous crushing force and displacement data were recorded directly using a data acquisition system.

### 2.3. Crashworthiness Indicators

To facilitate the crashworthiness design and performance evaluation of energy-absorbing structures, several critical indicators that could be calculated from the crushing force–displacement curves were selected based on previous studies [3,10]. These are the mean crushing force (MCF), energy absorption (EA), and specific energy absorption (SEA).

EA is defined as the energy dissipated through the collapse of the CFRP tubes, and can be determined as follows

(1) $$\mathrm{EA}=\int_{0}^{d}F\left(x\right)dx$$

where *F(x)* is the instantaneous crushing force at displacement *x*, and *d* is the total crushing distance. SEA denotes the absorbed energy per unit mass of the structure. It is defined as

(2) $$\mathrm{SEA}=\frac{\mathrm{EA}}{m}$$

where *m* denotes the mass of the crushed structure. MCF is the average crushing force during the crushing process, which is given by

(3) $$\mathrm{MCF}=\frac{\mathrm{EA}}{d}$$

### 2.4. Experimental Results and Discussion

**Figure 3:**
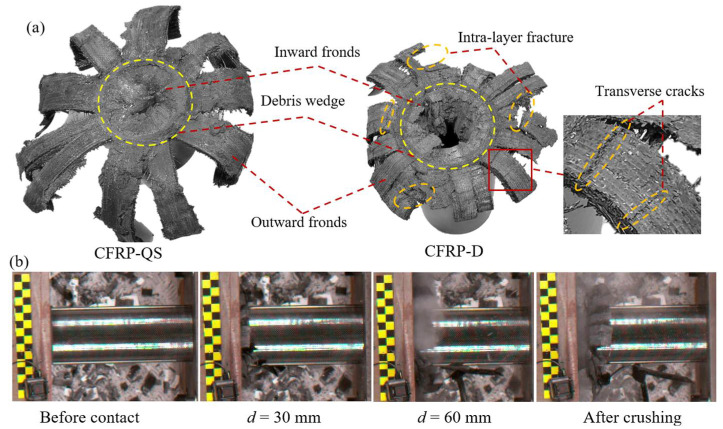
(**a**) Crushing failure modes and (**b**) crushing process of CFRP-D.

Figure 3a illustrates the failure modes of the CFRP specimens after the axial crushing tests. It can be observed that all specimens show a typical progressive end-crushing mode, which consists of a combination of transverse shear and lamina bending. This mode often appears for composite structures reinforced by brittle fiber materials as reported by Farley [57], which indicates a stable crushing process and load-carrying capability. When the CFRP tubes were crushed, longitudinal cracks were initiated at the chamfer side of the tube owing to the stress concentration, and which then propagated along the loading direction, splitting the CFRP tube wall into several pieces. As the crushing displacement increased, these fiber bundles were bent inward and outward along the middle line of the tube wall, leading to numerous damages among the curved fiber fronds, such as fragments, delamination, and longitudinal cracking (see Figure 3a). Moreover, owing to the friction and sliding between the loading platen and curved fiber fronds, a debris wedge was formed in the central area of the tube wall, which contributed to separating the tube wall into inward and outward fronds during the crushing process. In summary, the major energy absorption mechanisms of CFRP tubes in this study are similar to those of thin tubes [9], comprising the transverse shearing of fiber bundles, friction between the loading platen and curved fronds, and propagation of longitudinal and intra-wall cracks.

**Figure 4:**
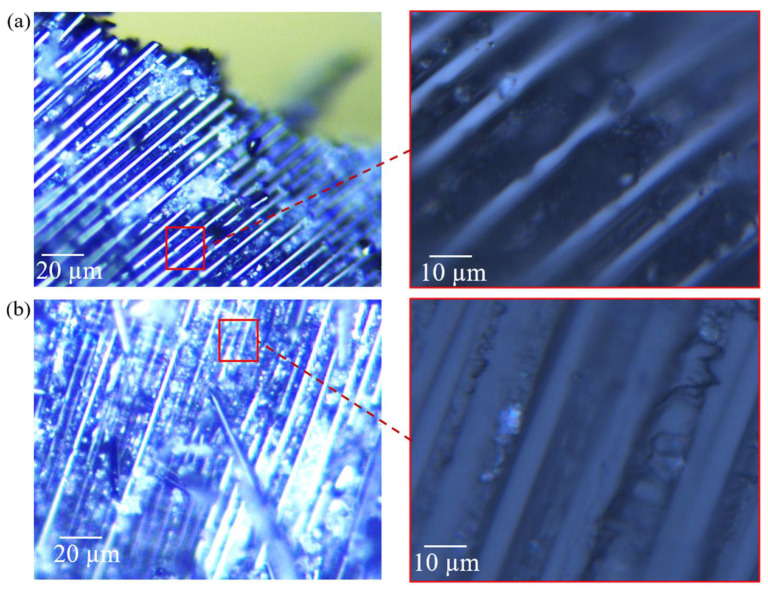
Partial images of crushed composite fragments for (**a**) CFRP-QS and (**b**) CFRP-D.

From Figure 3a, it is interesting to observe that CFRP-D has a weaker post-crash structural integrity than CFRP-QS, where larger amounts of transverse cracks were exhibited in the inward and outward fronds. This phenomenon correlates well with the test results reported in [58,59,60], where composite tubes with different shapes, such as double hat, circular, and half circular, were tested with a crushing velocity ranging from 2 mm/min to 12.36 m/s. Farley [58] reported that the mechanical properties of a matrix are often more sensitive to loading rates than brittle fibers (carbon fiber). Therefore, the complex failure behavior among crushed fronds, such as inter-layer crack growth, intra-layer matrix cracking, and debonding between the fibers and matrix phase, was significantly aggravated after the increase in loading velocity (Figure 4). Moreover, the friction coefficient between the loading platen and crushed fronds is typically sensitive to the impact velocity [39], which leads to the generation and ejection of numerous composite fragments during the crushing process (see Figure 3b).

**Figure 5:**
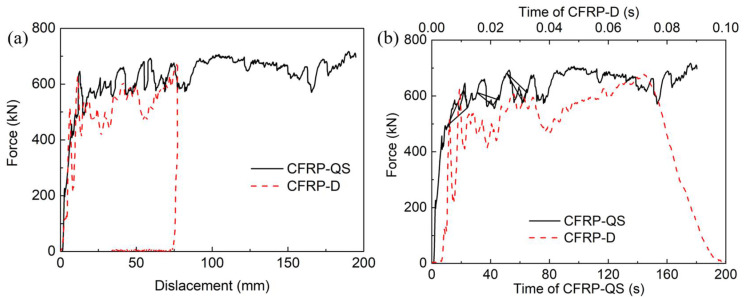
Comparison of axial crushing (**a**) force–displacement curves and (**b**) force–time curves.

The axial crushing force–displacement curves and force–time curves for CFRP tubes subjected to crushing velocities of 0.00108 m/s and 1.944 m/s are compared in Figure 5. These curves showed similar behavior, with a linear increase in the crushing force to a peak value at a displacement of 12 mm, which correlated well with the chamfer size. The peak force decreased to a plateau level owing to the stress concentration caused by the chamfer. Subsequently, a steady fluctuating force was obtained in the residual proportion, indicating a stable load-carrying process. Table 1 summarizes the crashworthiness indicators calculated from Figure 5, including the MCF, EA, and SEA. The MCF and SEA values of CFRP tubes crushed at 1.944 m/s are 485.7 kN and 78.7 kJ/kg, respectively, showing the same 21.8% reduction as those crushed at 0.00108 m/s. This phenomenon is similar to the results of double hat-shaped CFRP tubes in [29], where a reduction of 34.2% appeared after the loading velocity increased from 6 mm/min to 5 m/s. This can be attributed to the differences in the energy absorption mechanisms under different crushing velocities. A detailed analysis is presented in Section 3.4. In addition, SEA values for thin-walled CFRP tubes subjected to quasi-static loads often range between 50.4 kJ/kg and 86.3 kJ/kg [1,26,29,61], which are smaller than the values in this study.

Guan et al. [51] prepared circular metallic tubes (made of AISI 1020 steel) with similar geometrical dimensions to those of the CFRP tubes in this study. Axial crushing tests were performed at a velocity of 6.1 m/s. They found that the crushing energy was dissipated by the structural plastic deformation and friction between the dies and the tubal wall, which differed from the failure modes of CFRP tubes (see Figure 3). The MCF of the metallic tube was 432.4 kN, which was only 89% of the CFRP-D, and the SEA of the metallic tube was less than 20 kJ/kg, showing a minimum of 74.5% reduction. A similar phenomenon also appeared in [61], where the SEA values of thin-walled circular tubes made from aluminum, steel, and plain woven CFRP were reported as 13.2 kJ/kg, 10.0 kJ/kg, and 50.4 kJ/kg, respectively.

## 3. Numerical Simulations

### 3.1. Model Setup

**Figure 6:**
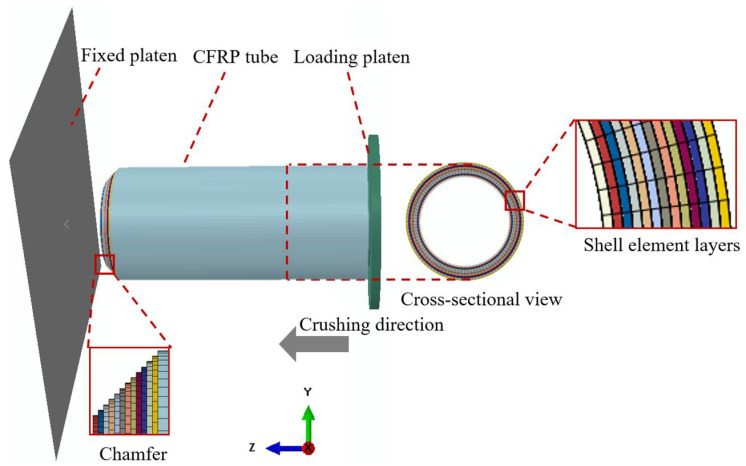
Numerical model of the axially crushing model.

Crashworthiness simulations of the composite tubes subjected to axial crushing loads were performed using the commercial software ABAQUS 6.13-4. Figure 6 illustrates the established FE model comprising a fixed platen, loading platen, and composite tube. The fixed and loading platens were separately set as rigid bodies with no deformation during the crushing process. The CFRP tubes were modeled using the classical stacked-shell strategy, where all the composite plies were represented using several layers of shell elements (S4) connected to the cohesive contact. A 45° chamfer was realized by adjusting the lengths of the different shell layers. As reported in the literature [31,46], a fine mesh size (less than 2 × 2 mm) and sufficient shell layers (equal to the real ply number) are required to simulate the crushing response of CFRP structures precisely. Because the ply number is up to 60 in this study, these practices could lead to unacceptable modeling and computational costs. Therefore, considering the influence of the number of shell layers is necessary, as described in Section 3.3.1.

During the crushing process, all degrees of freedom of the fixed platen were constrained, and only translation motion along the *z*-direction was allowed for the loading platen. To improve computational efficiency, the crushing velocity of the loading platen was defined as 2 m/s along the *z*-direction, and a mass scaling of 200 was applied for the crushing simulation of CFRP-QS. The feasibility of this strategy has been validated in a series of studies [10,32]. For the crushing simulation of CFRP-D, an initial velocity of 1.944 m/s and an impact mass of 18.19 t were applied to the loading platen. The initial length of the composite tubes was reduced to 150 mm in the quasi-static and dynamic simulations. A general contact algorithm was adopted to simulate the friction interaction between the composite tube and the two platens.

### 3.2. Constitutive Models

For the shell layers, a continuum damage mechanics-based material model was used to represent intra-layer failure behavior, such as fiber damage and matrix cracking [10,32]. In this model, the composite plies were modeled using a 2D orthogonal model. This model was implemented via the user-defined subroutine VUMAT in ABAQUS and has been sufficiently calibrated in previous studies [62]. A detailed description of this is provided in Appendix A. The material parameters used in the calculations are presented in Table 2.

Potential delamination failure (inter-layer damage) was modeled using a traction-separation law in conjunction with a surface-based cohesive contact model [31,63]. The quadratic nominal stress criterion embedded in ABAQUS was used to describe the damage initiation at the interfaces [10,64]

(4) $${\left\{\frac{\left\langle t_{n}\right\rangle }{t_{n}^{0}}\right\}}^{2}+{\left\{\frac{t_{s}}{t_{s}^{0}}\right\}}^{2}+{\left\{\frac{t_{t}}{t_{t}^{0}}\right\}}^{2}=1$$

where $t_{n}$, $t_{s}$, and $t_{t}$ denote the traction stress components along the normal, first, and second shear directions, respectively. $t_{n}^{0}$, $t_{s}^{0}$, and $t_{t}^{0}$ are the interface strengths in the corresponding directions, respectively. Once Equation (4) is satisfied, a linear degradation of interface properties is modeled using the damage evolution law

(5) $$G_{n}^{C}+\left(G_{s}^{C}-G_{n}^{C}\right){\left\{\frac{G_{s}+G_{t}}{G_{s}+G_{n}}\right\}}^{\eta }=G^{C}$$

where $G_{n}^{C}$, $G_{s}^{C}$, and $G^{C}$ are the critical fracture energies in the corresponding directions, respectively. $G_{n}$, $G_{s}$, and $G_{t}$ refer to the energy dissipated, and η denotes the mixed-mode coefficient. Table 2 also summarizes the required interface properties in the present simulation, which were extensively calibrated in [10,32,62].

In this study, the 60-ply composite tube was meshed into several layers of shell elements, indicating that each layer represents more than one composite ply. Cohesive contact was defined among the adjacent shell layers rather than between the composite plies. Because delamination occurs among composite ply interfaces (Figure 3), these practices fail to consider the additional delamination behavior, which tends to reduce predictive accuracy. Moreover, the tendency of instability is aggravated in axial crushing simulations of composite tubes [65]. Enhancing the interface properties is efficient in overcoming these drawbacks and has gained extensive attention [39,46,66]. Herein, the inter-layer fracture energies were increased and multiplied by a scaling factor *n* in the quasi-static and dynamic simulations. To provide a comprehensive comparison, *n* was defined as the ratio of the number of ply interfaces ($n_{p}$) to the number of cohesive interfaces ($n_{coh}$) or as its square root.

(6) $$n=\frac{n_{p}}{n_{coh}}$$

(7) $$n=\sqrt{\frac{n_{p}}{n_{coh}}}$$

### 3.3. Simulated Results and Validation

#### 3.3.1. Influences of the Number of Shell Layers

**Figure 7:**
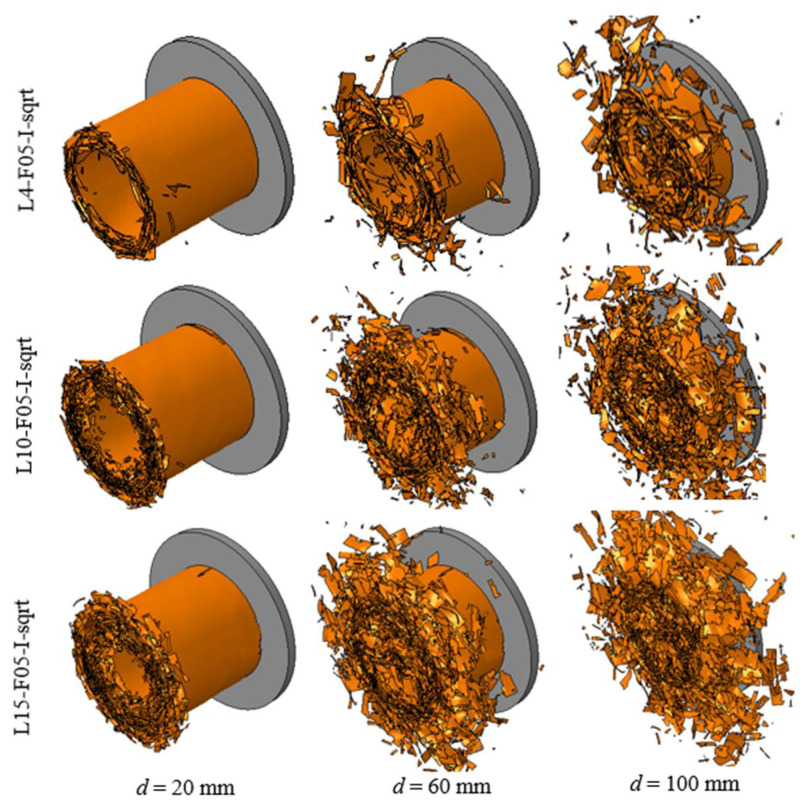
Simulated crushing process of the composite tube with shell layers of 4, 10, and 15.

As summarized in Table 3, three different models with 4, 10, and 15 layers of shell elements were established. In these analyses, the friction coefficient was set to 0.5, and the interface fracture energies were amplified using Equation (7). Figure 7 shows the predicted crushing response of the CFRP tube at crushing displacements of 20, 60, and 100 mm, respectively. It can be observed that the collapse modes predicted using 10 and 15 layers were closer to the tested mode than those predicted using 4 layers.

**Figure 8:**
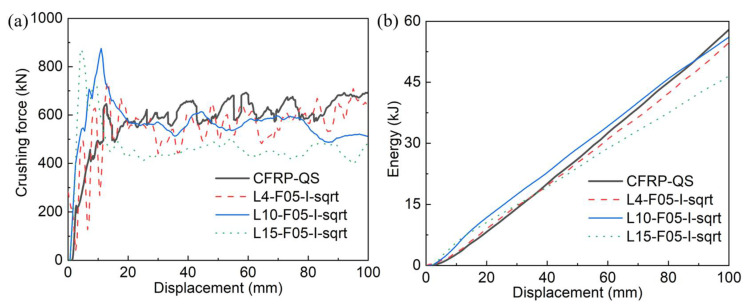
Comparisons of (**a**) crushing force–displacement curves and (**b**) energy–displacement curves between experimental (CFRP-QS) and simulated results with shell layers of 4, 10, and 15.

Figure 8 illustrates the comparison of the crushing force–displacement and energy–displacement curves between the simulated and tested results, while Table 3 summarizes the predicted energy absorption and its error compared to the experimental (CFRP-QS) value. The maximum error that appeared for the EA was approximately 19.7%, where 15 layers of shell elements were adopted during the simulation (L15-F05-I-sqrt). Crushing models using 4 and 10 layers of shell elements showed good accuracy with maximum errors of 5.5% and 3.3%, respectively. This is an interesting phenomenon that also exists in Reference [46] and can be attributed to differences in the failure modes.

#### 3.3.2. Influences of Friction Coefficient

**Figure 9:**
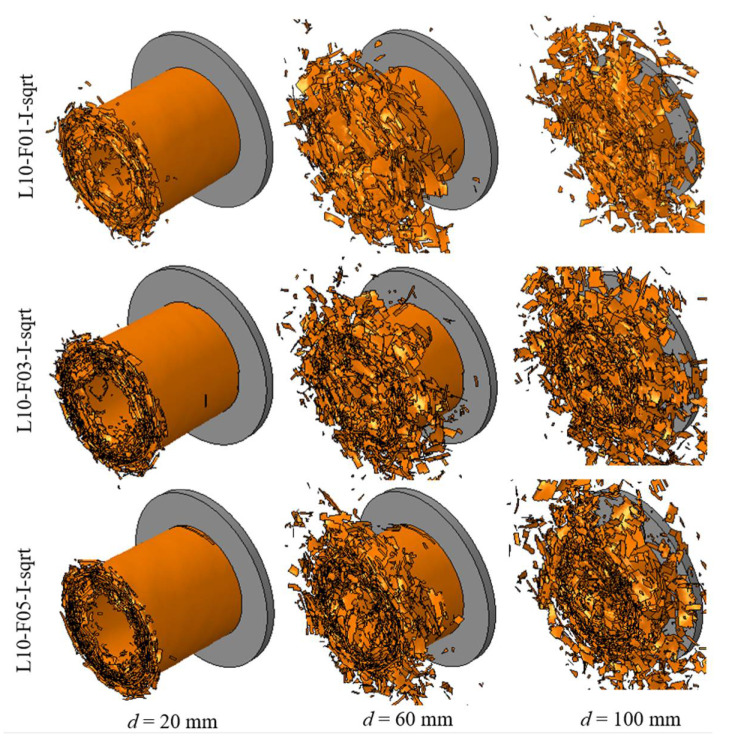
Simulated crushing process of the composite tube with friction coefficients of 0.1, 0.3, and 0.5.

**Figure 10:**
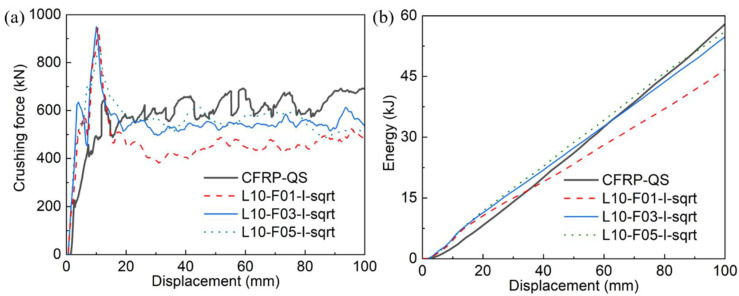
Comparisons of (**a**) crushing force–displacement curves and (**b**) energy–displacement curves between experimental (CFRP-QS) and simulated results with friction coefficients of 0.1, 0.3, and 0.5.

The effect of the friction coefficient was studied using a validated FE model with 10 layers of shell elements. Three different friction coefficient values (0.1, 0.3, and 0.5) were examined. Figure 9 and Figure 10 present the predicted results, including the crushing process, force–displacement, and energy–displacement curves. It can be observed that all the curves show a trend similar to that of the test curve, and the predicted collapse modes are quite close to the tested mode (see Figure 3a).

Table 3 lists the energy absorption data obtained from each simulation and their error relative to the experimental value. It can be observed that the maximum error for EA, between the simulated and tested values, was 19.5% (L10-F01-I-sqrt). Increasing the friction coefficient from 0.1 to 0.5 decreased this error to 3.3%. This can be attributed to the enhancement in the friction interaction between the loading platen and curved fiber bundles.

#### 3.3.3. Influences of Interface Properties

The composite tube was modeled using 10 layers of shell elements with the same friction coefficient of 0.5. Three different pairs of values for the inter-layer fracture energies $G_{n}^{C}$ and $G_{s}^{C}/G^{C}$ were examined, as shown in Table 3. The lowest values of $G_{n}^{C}$ and $G_{s}^{C}/G^{C}$ (L10-F05-I-no) are the original parameters listed in Table 2. For the other two tested configurations, the interface properties were amplified using scaling factors calculated using Equation (6) and Equation (7), respectively.

**Figure 11:**
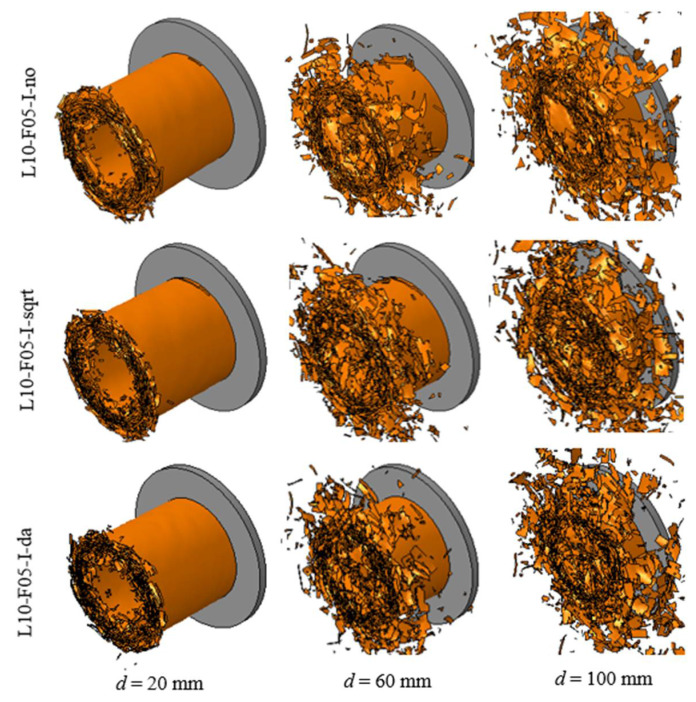
Crushing process of the composite tube simulated using three pairs of inter-layer fracture energy values.

**Figure 12:**
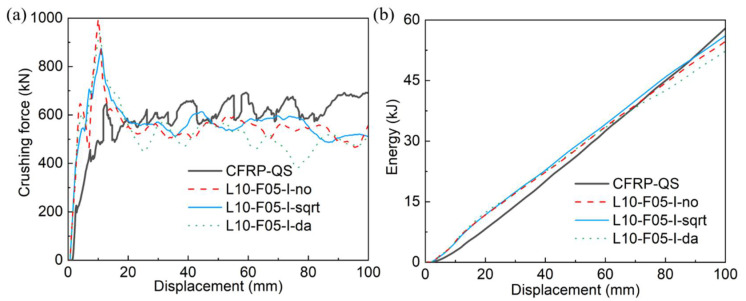
Comparisons of (**a**) crushing force–displacement curves and (**b**) energy–displacement curves between experimental (CFRP-QS) and simulated results using three pairs of inter-layer fracture energy values.

Figure 11 and Figure 12 show the crushing process, force–displacement, and energy–displacement curves of the composite tube, which were predicted using different interface fracture energies. All the crushed composite tubes exhibited a stable collapse mode. When the initial inter-layer fracture energy values (L10-F05-I-no) were adopted, large amounts of delamination and intra-layer lamina fractures occurred. Increasing these parameters using Equation (7) (L10-F05-I-sqrt) reduced the geometrical sizes of the fractured fiber bundles, and led to a minor increase in EA from 54.6 kJ to 56.0 kJ. However, a larger error of 9.7% appeared after the interface fracture energies were increased using Equation (6).

#### 3.3.4. Dynamic Simulation Results

**Figure 13:**
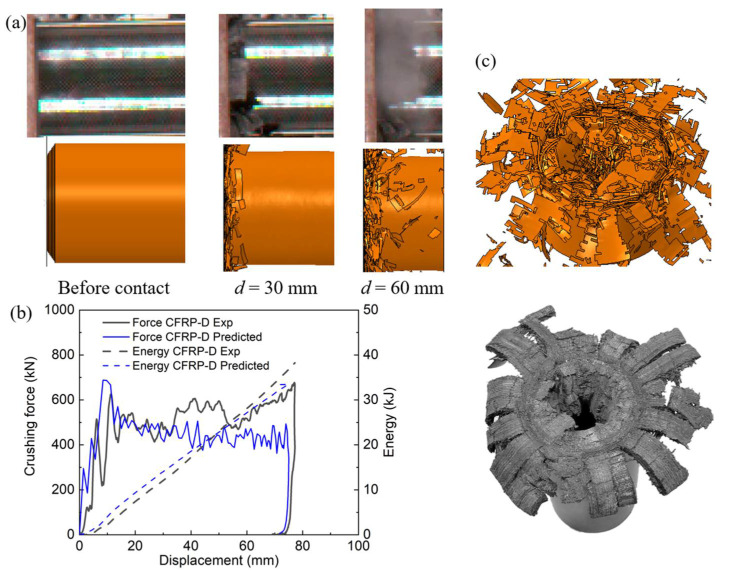
Comparison of the experimental and simulated results of the composite tube: (**a**) crushing process, (**b**) crushing force–displacement and energy–displacement curves, and (**c**) failure modes.

The crushing response of composite tubes crushed by a trolley was investigated. For this analysis, the shell layer was set to 10, and the interface fracture energies were amplified using Equation (7). The friction coefficient value was set as 0.3, which was reported to be smaller than the values under static loads [39]. Comparisons of the crushing process, force–displacement and energy–displacement curves, and failure modes between the experimental and simulated results are presented in Figure 13. It can be observed that the simulated crushing process agrees well with the tested results, including the initiation of composite damage on one side of the circular tube and propagation along the loading direction, together with the ejection of numerous fiber debris. The EA values from the dynamic crushing experiment and simulation were 37.5 kJ and 33.4 kJ, respectively, resulting in an error of 10.9%. The predicted curves also show good agreement, indicating that the established FE model is capable of capturing the crushing response of the CFRP tubes.

### 3.4. Energy-Absorbing Mechanisms’ Analysis

**Figure 14:**
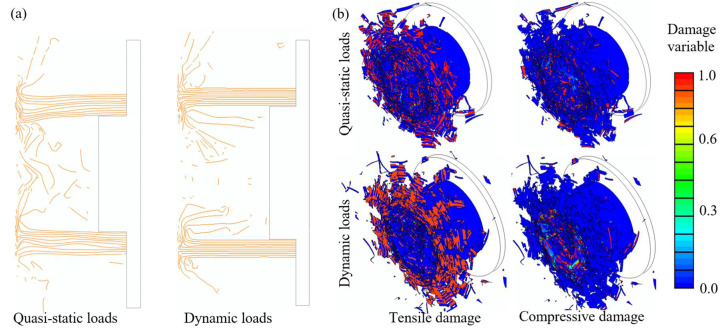
Failure modes of composite tubes after axial crushing tests: (**a**) cross-sectional view (each line refers to a shell layer) and (**b**) distribution of tensile and compressive damage variables.

**Figure 15:**
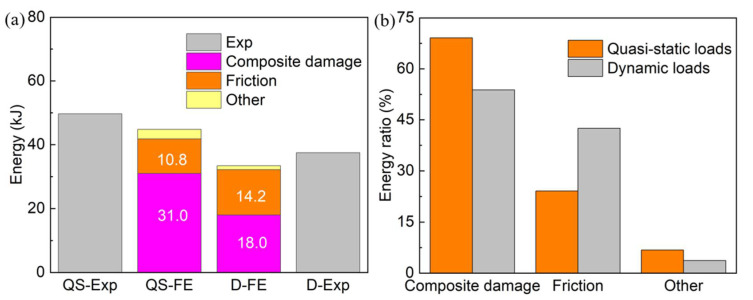
Comparisons of (**a**) energy components and (**b**) their proportion to total energy absorption of composite tubes subjected to quasi-static (QS) and dynamic (D) loads.

This section aims to explore the influence of crushing velocity on the energy-absorbing mechanisms of CFRP tubes. A comprehensive comparison of the distribution of the composite damage variables, cross-sectional views, and energy components is illustrated in Figure 14 and Figure 15. In a dynamic impact event, the crushed displacement of the loading platen was 77.2 mm, which differs from the 195 mm under quasi-static loads. To facilitate the comparison analysis, the quasi-static simulated results were obtained from the validated FE model of L10-F05-I-sqrt at the same crushing displacement of 77.2 mm.

Figure 14a depicts a cross-sectional view of the composite tubes subjected to quasi-static and dynamic impact loads. A similar deformation mode was achieved from these two simulations: large amounts of fiber debris appeared in the central area between the loading platen and tube wall, and the crushed laminas were bent inward and outward of the tube wall along the loading direction. The curved fiber bundles suffered complicated deformation and damage processes, such as inter-layer delamination and tensile/compressive damage behavior (illustrated in Figure 14b). Increasing the loading velocity promoted a more stable load-carrying capability, which agreed well with the test results, as shown in Figure 3. 

Figure 15 illustrates the comparison of different energy components among the quasi-static and dynamic simulations, including composite damage energy, friction energy, and others (caused by the calculation error of the FE software). It can be observed that the energies dissipated by the composite damage and friction effect were 31.0 kJ and 10.8 kJ, respectively, under quasi-static loads. These were 69.1% and 24.1% of the total energy, respectively. When the loading velocity increased, the composite damage energy and friction energy changed to 18.0 kJ and 14.2 kJ, which were 53.8% and 42.5% of the total energy, respectively. This change explains the influence of the loading velocity on the reduction in total EA.

## 4. Conclusions

The quasi-static and dynamic axial crushing responses of thick circular CFRP tubes were investigated using experimental and numerical approaches. In the experimental studies, the crushing modes, force–displacement curves, and crashworthiness parameters were explored and compared. A numerical model was developed in ABAQUS to simulate the crushing behavior of CFRP tubes subjected to both loading scenarios. The stacked-shell modeling strategy was adopted, wherein the intra-layer behavior was modeled using several shell layers, and inter-layer delamination was modeled using cohesive contact. The influences of several parameters, such as the number of shell layers, friction coefficient, and interface properties, were analyzed and compared. Finally, the energy-absorbing mechanisms of the circular CFRP tubes were analyzed and compared in detail. The main conclusions are as follows:

(1) Under quasi-static and dynamic axial impact loads, the circular CFRP tubes exhibited a stable crushing response. However, the values of MCF and SEA under dynamic crushing loads were 485.7 kN and 78.7 kJ/kg, respectively, showing the same 21.8% reduction compared to the quasi-static cases.
(2) A parametric study on modeling strategies showed that the predicted EA was sensitive to a series of modeling parameters, including the number of shell layers, friction coefficient, and interface properties. A small variation in these parameters can have a negligible influence on the failure mode but a significant change in the total EA.
(3) The validated FE models showed good agreement in predicting the crushing response of circular CFRP tubes. The errors in crashworthiness indicators, such as MCF and SEA, were less than 10%.
(4) Composite damage and friction interaction accounted for a major portion of the energy-absorbing behavior: their percentages in the EA were 69.1% and 24.1%, respectively, under quasi-static crushing loads. Dynamic crushing loads resulted in a 41.9% reduction in composite damage energy but a 31.4% increase in friction energy, resulting in a reduction in SEA compared to the quasi-static cases.

The presented results demonstrate the feasibility of CFRP composite tubes for energy-absorbing structures that require a large load-carrying capability. Considering their high material cost and poor post-crush integrity, future work will focus on (i) hybridization using ductile fibers with a lower material cost and (ii) introducing triggering devices.

## Figures and Tables

**Table 1:** Geometrical and crashworthiness parameters for circular CFRP tubes.

Test Code	*t*/mm	*D*/mm	*L*/mm		Mass/kg	MCF/kN	EA/kJ	SEA/kJ/kg
			Initial	Crushed				
CFRP-QS	12.4	100	300	195	1.87	621.5	121.2	99.7
CFRP-D	12.4	100	300	77.2	1.85	485.7	37.5	78.7

**Table 2:** Material properties used in simulations.

Property	Values	Property	Values
$E_{11}/E_{22}$ (GPa)	59.2 [62]	$t_{n}^{0}$ (MPa)	54 [32]
$G_{12}$ (GPa)	3.7 [62]	$t_{s}^{0}/t_{t}^{0}$ (MPa)	70 [32]
$\upsilon_{12}$	0.067 [62]	$G_{n}^{C}$ (J/m^2^)	504 [62]
$X_{1t}/X_{2t}$ (MPa)	679.1 [10]	$G_{s}^{C}/G_{t}^{C}$ (J/m^2^)	1566 [62]
$X_{1c}/X_{2c}$ (MPa)	512 [10]	η	2.284 [32]
$S_{12}$ (MPa)	56.1 [10]	/	/
$G_{1t}^{f}/G_{2t}^{f}$ (kJ/m^2^)	155 [62]	/	/
$\varepsilon_{1c}^{f}/\varepsilon_{2c}^{f}$	0.36 [62]	/	/

**Table 3:** Comparisons of EA between tests and simulations with different configurations for CFRP-QS.

Code	Layer Number	Friction Coefficient	Amplification of Interface Properties	Predicted EA (kJ)	Error for EA (%)
L4-F05-I-sqrt	4	0.5	Using Equation (7)	54.7	−5.5
L10-F05-I-sqrt	10	0.5	Using Equation (7)	56.0	−3.3
L15-F05-I-sqrt	15	0.5	Using Equation (7)	46.5	−19.7
L10-F01-I-sqrt	10	0.1	Using Equation (7)	46.6	−19.5
L10-F03-I-sqrt	10	0.3	Using Equation (7)	54.7	−5.5
L10-F05-I-no	10	0.5	None	54.6	−5.7
L10-F05-I-da	10	0.5	Using Equation (6)	52.3	−9.7

Note: A labelling system was adopted to facilitate analysis. For example, L10-F05-I-sqrt indicates the model using 10 layers (L) of shell elements, a friction (F) coefficient of 0.5, and interface fracture energies amplified by a coefficient calculated using Equation (7). This calculation was adopted as a reference for comparing the results predicted using each variable group.

**Figure A1:**
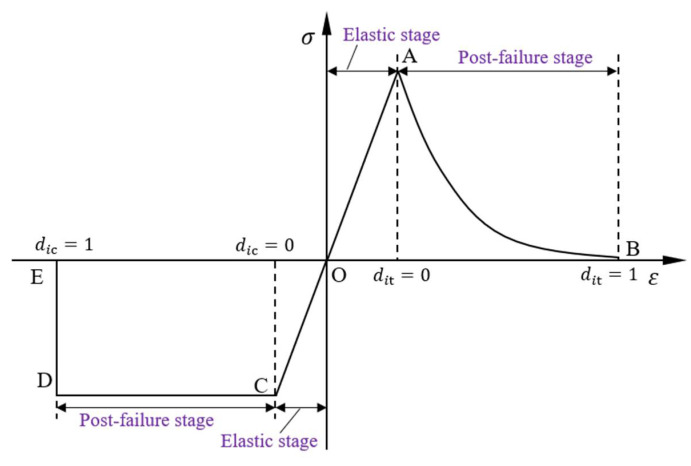
Schematic view of the stress–strain curve for intra-layer material.

## Data Availability

The data that support the findings of this study are available within the article.

## Appendix A. Constitutive Laws for Intra-Layer Material

A two-dimensional (2D) constitutive law was adopted to simulate the mechanical behavior of the plain woven fabric reinforcement. Figure A1 illustrates the stress–strain relationships under tensile or compressive loads, which mainly comprise the linear elastic stage (OA/OC) and post-failure stage (AB/CD). The in-plane stress–strain constitutive relationship is then formulated as [32]

(A1) $$\left\{\begin{array}{c} \varepsilon_{11}\\ \varepsilon_{22}\\ \varepsilon_{12} \end{array}\right\}=\left[\begin{array}{ccc} \frac{1}{\left(1-d_{1}\right)E_{11}} & -\frac{\upsilon_{12}}{E_{11}} & 0\\ -\frac{\upsilon_{21}}{E_{22}} & \frac{1}{\left(1-d_{2}\right)E_{22}} & 0\\ 0 & 0 & \frac{1}{\left(1-d_{12}\right)2G_{12}} \end{array}\right]\left\{\begin{array}{c} \sigma_{11}\\ \sigma_{22}\\ \sigma_{12} \end{array}\right\}\;$$

where 11, 22, and 12 represent the longitudinal fiber, transverse fiber, and in-plane shear directions, respectively. $\sigma_{ij}$ and $\varepsilon_{ij}$ (i,j=1,2) denote the stress and strain components, respectively. $E_{11}$ and $E_{22}$ are the Young’s moduli along the longitudinal and transverse directions, respectively, and $G_{12}$ is the in-plane shear modulus. $\upsilon_{12}$ and $\upsilon_{21}$ refer to Poisson’s ratios. The damage variables, $d_{1}$, $d_{2}$, and $d_{12}$, were set to zero in the linear elastic stage. The failure criteria used to determine the damage initiation of the different failure modes (points A and C in Figure A1) were defined according to previous studies [62] as follows:

Longitudinal/transverse failure in tension/compression

(A2) $$F_{1t}=\frac{\sigma_{11}}{X_{1t}}-r_{1t},\;\varepsilon_{11}>0$$

(A3) $$F_{1c}=\frac{\sigma_{11}}{X_{1c}}-r_{1c},\;\varepsilon_{11}<0$$

(A4) $$F_{2t}=\frac{\sigma_{22}}{X_{2t}}-r_{2t},\;\varepsilon_{22}>0$$

(A5) $$F_{2c}=\frac{\sigma_{22}}{X_{2c}}-r_{2c},\;\varepsilon_{22}<0$$

(A6) $$r_{\alpha }\left(t\right)=\underset{\tau \leq t}{\max}\frac{\sigma_{\alpha }}{X_{\alpha }}\left(\tau \right),\;(\alpha \;=\;1t,\;1c,\;2t\;\mathrm{and}\;2c)$$

where $F_{\alpha }$ and $X_{\alpha }$ denote the failure factors and material strength parameters under tensile (*t*) and compressive (*c*) loads along the longitudinal and transverse directions, respectively. $r_{\alpha }$ are the damage thresholds and are initially set to 1.0. Once $F_{\alpha }$ is equal to 1.0 (failure criteria are met), the damage variables can be updated following Equations (A6) and (A7) [32]

(A7) $$d_{\alpha }=\{\begin{array}{c} 1-\frac{1}{r_{\alpha }}exp\left[-\frac{2g_{\alpha }^{0}l_{c}}{G_{\alpha }^{f}-g_{\alpha }^{0}l_{c}}\left(r_{\alpha }-1\right)\right],\;\mathrm{if}\;\varepsilon_{\alpha }>0\;\\ \frac{\left|\varepsilon_{\alpha }\right|-X_{\alpha }/E_{\alpha }}{\left|\varepsilon_{\alpha }^{f}\right|-X_{\alpha }/E_{\alpha }},\;\mathrm{if}\;\varepsilon_{\alpha }\leq 0\; \end{array}$$

where g$g_{\alpha }^{0}$ and $G_{\alpha }^{f}$ denote the elastic energy density and fracture energy per unit area, respectively, under the different loading modes. $l_{c}$ is the characteristic element length. $\varepsilon_{\alpha }^{f}$ represents the ultimate strain under compressive loading.
